# The prevalence and mechanism of fluoroquinolone resistance in *Escherichia coli* isolated from swine farms in China

**DOI:** 10.1186/s12917-020-02483-4

**Published:** 2020-07-28

**Authors:** Ping Cheng, Yuqi Yang, Fulei Li, Xiaoting Li, Haibin Liu, Saqib Ali Fazilani, Wenxin Guo, Guofeng Xu, Xiuying Zhang

**Affiliations:** 1 Heilongjiang Key Laboratory for Animal Disease Control and Pharmaceutical Development. Faculty of Basic Veterinary Science, College of Veterinary Medicine, Northeast Agricultural University, 600 Changjiang Road, Xiangfang District, Harbin, Heilongjiang 150030 P.R. China; 2 Pharmacology Teaching and Research Department, School of Basic Medicine, Guizhou University of Traditional Chinese Medicine, Dongqing Road, University Town, Huaxi District, Guiyang, P.R. China; 3 Heilongjiang Technical Identification Station of Agricultural products and Veterinary Medicine Feed, Harbin, China; 4 Inflammation & Allergic Diseases Research Unit, Affiliated Hospital of Southwest Medical University, Luzhou, 646000 Sichuan China

**Keywords:** Fluoroquinolone resistance, *Escherichia coli*, Swine, PMQR, Target mutations

## Abstract

**Background:**

It has been demonstrated that swine waste is an important reservoir for resistant genes. Moreover, the bacteria carrying resistant genes and originating from swine feces and wastewater could spread to the external environment. Fluoroquinolones (FQs) are widely used in livestock and poultry for the treatment of bacterial infection. However, resistance to FQs has increased markedly.

**Results:**

In this study, swine feces and wastewater were sampled from 21 swine farms of seven provinces in China to investigate the prevalence of FQ resistance, including plasmid-mediated fluoroquinolone resistance (PMQR) genes and the occurrence of target mutations. All isolates showed moderate rate of resistance to norfloxacin (43.0%), ciprofloxacin (47.6%), ofloxacin (47.0%) and levofloxacin (38.8%). The percentage of strains resistant to the four FQs antimicrobials was positively correlated with the danofloxacin (DANO) MIC. Among the 74 FQ-resistant isolates, 39 (52.70%) had mutations in *gyrA* (S83L and D87 to N, Y, G, or H), 21 (28.38%) had mutations in *parC* (S80I and E84K), 2 (2.70%) had mutations in *parE* (I355T and L416F), 26 (35.14%) had mutations in *marR* (D67N and G103S), 1 (1.35%) had mutations in *acrR* (V29G). While, no mutation was found in *gyrB*. There were 7 (9.46%) strains carried the *qnrS* gene, 29 (39.19%) strains carried the *oqxAB* gene, and 9 (12.16%) strains carried the *aac (6′)-Ib-cr* gene. In addition, the conjugation assays showed that *qnrS*, *oqxAB* and *aac (6′)-Ib-cr* could be successfully transferred to *E. coli* J53 from 4 (57.1%), 20 (69.0%) and 5 (55.6%) donor strains, respectively. There were no *qnrA*, *qnrB*, *qnrC*, *qnrD* and *qepA* genes detected.

**Conclusion:**

The present study showed that DANO-resistant *E. coli* strains isolated from swine farms had significant cross-resistance to other four FQs antimicrobials. Further study revealed that the resistance mechanisms of swine-derived *E. coli* to FQs may be attributable to the occurrence of chromosomal mutations (*gyrA*, *parC*, *parE*, *marR* and *acrR* genes double-site or single-site mutation) and the presence of PMQR genes (*qnrS, oqxAB and aac (6′)-Ib-cr*). To the best of our knowledge, one novel mutation *marR*-D67N was found to be associated with FQ resistance, two mutations *parE*-L416F and *acrR*-V29G have never been reported in China.

## Background

Antimicrobial resistance has posed an imminent threat to global health, which threatens our ability to treat common infections caused by bacterial organisms [1]. Due to the restricted choice of antimicrobials as well as the dearth of novel classes of antimicrobials emergence, the infections caused by multidrug-resistant bacteria (MRB) are often accompanied by high morbidity and mortality [2]. It is now generally accepted that the emergence and prevalence of MRB is associated with the widespread, unreasonable, and increasing use of antimicrobials. This could raise a potential risk for the selection of bacteria to become resistant and promote the dissemination of antimicrobial-resistant bacteria (ARB) and resistance genes [3].

Animals are regarded as an important reservoir of resistance genes or ARB which could cause bacterial infection in humans [4]. It has been demonstrated that the possibility for transfer of ARB between animals and humans through environment, food chains and direct contact. Therefore, the emergence of ARB originated from animals have become a growing area of concern. As a common member and the most prevalent enteric bacteria in the intestinal tract of animals and humans, *E. coli* can also be associated with animal and human infectious diseases due to their zoonotic potential [5, 6]. Hence, the level of resistance in commensal *E. coli* is regarded as a good indicator for potential selection pressure exerted by regular use of antimicrobials and for investigation of resistance problems in pathogenic bacteria [3, 7].

As a series of synthetic and broad-spectrum antimicrobial agents, FQs possess the bactericidal activity, which can prevent the bacterial cell growth by inhibiting the activities of DNA gyrase and DNA topoisomerase IV, interfering with DNA replication, recombination and repair. Moreover, with the advantages of high bioavailability and low incidence of adverse effects, FQs are widely used in the treatment of a variety of bacterial infections and parasitic diseases [8, 9]. The purpose of promoting the economic benefits by limiting the mortality of animals, improving feed efficiency and stimulating the uniformity between animals has resulted in the increase use of FQs antimicrobials in farms with the inevitable risk of emerging resistance [10].

FQ resistance in enterobacteriaceae was commonly thought to be chromosome-mediated through mutations in the genes encoding DNA gyrase (*gyrA* and *gyrB*), topoisomerase IV (*parC* and *parE*) and the operons of endogenous transmembrane efflux pump AcrAB-TolC (*marR* and *acrR*), until the PMQR was found in 1998 [11, 12]. The mutations in *gyrA*, *gyrB*, *parC* and *parE* associated with FQ resistance are often located in a region known as the fluoroquinolone resistance determining region (QRDR), which can decrease the affinity of the mutant enzyme-DNA complex to FQs antimicrobials [13]. The mutations in *marR* and *acrR* can accelerate the efflux of FQs antimicrobials from the bacterial cytoplasm by increasing the expression of AcrAB-TolC [11]. As alternative mediators of FQ resistance, PMQR was first reported in *Klebsiella pneumoniae* [12]. Importantly, five major groups of Qnr determinants (*QnrA*, *QnrB*, *QnrC*, *QnrD*, and *QnrS*) that encode DNA gyrase protection proteins were identified, which were regarded to have the potential to reduce the susceptibility to FQs and lead to resistance. Efflux pumps *(oqxAB* and *qepA*) and a variant of aminoglycoside-modifying enzyme *aac (6′)-Ib-cr* were proven to be two additional PMQR determinants in previous study [14, 15].

The aims of this study were to examine the susceptibility of *E. coli* strains isolated from several swine farms in China to norfloxacin, ciprofloxacin, ofloxacin and levofloxacin, to analyze the correlation between the susceptibility of the strains to the four FQs antimicrobials and the resistance to DANO, to investigate the prevalence of PMQR genes (*qnrA, qnrB, qnrS, qepA, oqxAB, qnrC, qnrD* and *aac (6′)-Ib-cr*) and the occurrence of chromosomal mutations in *gyrA*, *gyrB*, *parC*, *paE, marR* and *acrR* among the FQ-resistant *E. coli* strains.

## Results

From July 2014 to March 2017, a total of 1222 *E. coli* were recovered from swine feces (1000) and wastewater (222). Isolates were collected from Heilongjiang (*n* = 404), Jilin (*n* = 263), Liaoning (*n* = 227), Henan (*n* = 249), Shandong (*n* = 30), Hubei (*n* = 20), and Yunnan (*n* = 29) provinces of China. In this study, a total of 479 *E. coli* isolates were selected for further study, which cover the MIC distribution range (0.0075- > 128 μg/mL) of DANO. (as shown in Table S1).

### Comparisons of antimicrobial resistance in isolates

**Fig. 1:**
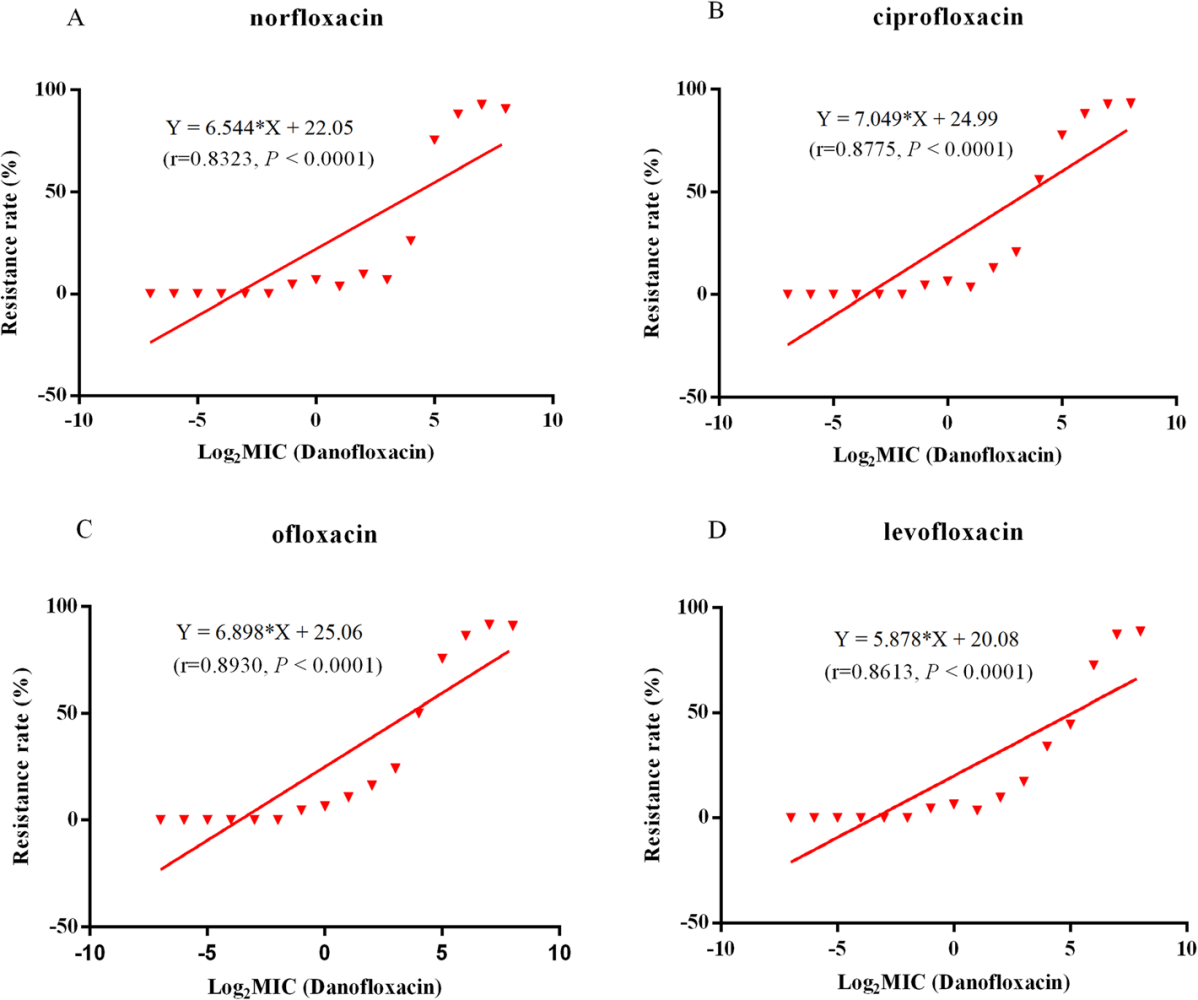
Correlation between the resistance rate of *E. coli* to four FQs antimicrobials and DANO MICs

The results of the antimicrobial susceptibility testing were shown in Table 1, all isolates showed moderate rate of resistance to norfloxacin (43.0%), ciprofloxacin (47.6%), ofloxacin (47.0%) and levofloxacin (38.8%). As shown in Fig. 1, the percentage of strains resistant to ciprofloxacin (*r* = 0.8775, *P* < 0.0001), ofloxacin (*r* = 0.8930, *P* < 0.0001), levofloxacin (*r* = 0.8613, *P* < 0.0001) and norfloxacin (*r* = 0.8323, *P* < 0.0001) was positively correlated with the degree of resistance to DANO.

**Table 1:** Prevalence of resistance to FQs antimicrobials in swine-derived *E. coli* isolates of different DANO MICs

Antimicrobials	DANO MIC (μg/mL)	HLJ	JL	LN	SD	HN	HB	YN	Resistance (%)	Total
Norfloxacin	≥128	27/34	26/27	24/24		26/27	1/1	1/1	92.11% (105/114)	
	64	13/16	11/14	11/11		10/10			88.24% (45/51)	
	32	9/15	8/12	10/10	1/2	6/6			75.56% (34/45)	
	16	1/10	1/10	1/10	7/10	3/10			26% (13/50)	
	8^a^	0/7	0/6	2/7		0/7		0/2	6.9% (2/29)	
	4–0.5	4/24	0/18	2/20	1/6	0/18	0/15	0/11	6.25% (7/112)	
	≤0.25	0/20	0/17	0/14	0/3	0/18	0/4	0/2	0 (0/78)	43.00% (206/479)
Ciprofloxacin	≥128	28/34	26/27	24/24		26/27	1/1	1/1	92.98% (106/114)	
	64	13/16	11/14	11/11		10/10			88.24% (45/51)	
	32	10/15	8/12	10/10	1/2	6/6			77.78% (35/45)	
	16	1/10	5/10	5/10	10/10	7/10			56% (28/50)	
	8^a^	0/7	1/6	4/7		1/7		0/2	20.69% (6/29)	
	4–0.5	4/24	1/18	2/20	1/6	0/18	0/15	0/11	7.14% (8/112)	
	≤0.25	0/20	0/17	0/14	0/3	0/18	0/4	0/2	0 (0/78)	47.60% (228/479)
Ofloxacin	≥128	26/34	26/27	24/24		26/27	1/1	1/1	91.23% (104/114)	
	64	12/16	12/14	10/11		10/10			86.27% (44/51)	
	32	10/15	8/12	9/10	1/2	6/6			75.56% (34/45)	
	16	1/10	3/10	4/10	10/10	7/10			50% (25/50)	
	8^a^	0/7	1/6	3/7		3/7		0/2	24.14% (7/29)	
	4–0.5	4/24	1/18	4/20	1/6	1/18	0/15	0/11	9.82% (11/112)	
	≤0.25	0/20	0/17	0/14	0/3	0/18	0/4	0/2	0 (0/78)	47.00% (225/479)
Levofloxacin	≥128	24/34	25/27	23/24		26/27	1/1	1/1	87.72% (100/114)	
	64	9/16	9/14	9/11		10/10			72.55% (37/51)	
	32	3/15	7/12	4/10	1/2	5/6			44.44% (20/45)	
	16	1/10	0/10	2/10	9/10	5/10			34% (17/50)	
	8^a^	0/7	0/6	3/7		2/7		0/2	17.24% (5/29)	
	4–0.5	4/24	0/18	2/20	1/6	0/18	0/15	0/11	6.25% (7/112)	
	≤0.25	0/20	0/17	0/14	0/3	0/18	0/4	0/2	0 (0/78)	38.83% (186/479)

Note: *DANO* Danofloxacin, *HLJ* Heilongjiang, *JL* Jilin, *LN* Liaoning, *SD* Shandong, *HN* Henan, *HB* Hubei, *YN* Yunan; ^a^, the susceptibility breakpoint of DANO against swine-derived *E. coli* [5]

A total of 74 *E. coli* strains resistant to the four FQs antimicrobials were selected for further study to determine the prevalence of PMQR genes and the occurrence of target mutations in QRDR.

### Detection of Fluoroquinolone resistance-associated mutations

As shown in Tables 2 and 3, among the 74 *E. coli* isolates, no mutations were found in *gyrB*. Alterations in the *gyrA* gene were detected in 39 (52.7%) of the 74 *E. coli* isolates. There were five types of mutations observed in the FQ resistance determining region (QRDR) of *gyrA*: 32 (82.1% of the 39 *gyrA* mutated isolates) isolates had S83L and D87N, 2 (5.1%) had S83L and D87Y, 1 (2.6%) had S83L and D87H, 1 (2.6%) had S83L and D87G, as well as 3 (7.7%) had S83L. Twenty-one strains (28.4%) carried mutations in the QRDR of *parC*: 18 strains (85.7%) had S80I, 2 (9.5%) had S83L and E84K and 1 (4.8%) had E84K. Twenty-six strains (35.1%) carried mutations in *marR*: 25 strains (96.2%) had G103S and 1 (3.8%) had D67N and G103S. Two strains (2.7%) carried double *parE* mutations as: I355T and L416F. One strain carried mutation in *acrR*, the mutation was V29G. The original dataset was provided in Table S2, and the Gel electrophoresis of the positive PCR product was provided in Figure S1.

**Table 2:** The Sorting Intolerant From Tolerant (SIFT) scores of different mutations

	Topoisomerases and gyrase mutations									Efflux-related mutations		
Genes	*gyrA*					*parC*		*parE*		*marR*		*acrR*
Sites	83	87	87	87	87	80	84	355	416^b^	67^a^	103	29^b^
Amino acid alterations	S	D	D	D	D	S	E	I	L	D	G	V
	L	N	Y	G	H	I	K	T	F	N	S	G
Proven Scores	−4.2	−4.6	−8.3	−6.5	−6.3	−5.8	−3.9	2.9	−3.9	−4.4	1.4	−6.3
Prediction	DT	DT	DT	DT	DT	DT	DT	NT	DT	DT	NT	DT

Note: Score thresholds for prediction: Default threshold is −2.5, variant with a score equal to or below −2.5 are considered “deleterious”, variant with a score above −2.5 are considered “neutral”; *QRDR* fluoroquinolone resistance determining region, *DT* Deleterious, *NT* Neutral; ^a^ The novel mutation was found to be associated with fluoroquinolone resistance; ^b^The mutation has never been reported in China

**Table 3:** Putative FQ resistance mutations and mutation rate

NO of isolates and DANO MIC (μg/mL)	Topoisomerases and gyrase mutations			Efflux-related mutations	
	*gyrA*	*parC*	*parE*	*marR*	*acrR*
13 (128); 2 (64);	S83L, D87N	None	None	None	None
2 (128); 1 (64)	S83L, D87N	S80I	None	None	None
5 (128); 3 (64)	S83L, D87N	S80I	None	G103S	None
2 (128); 1 (32)	S83L, D87N	None	None	G103S	None
2 (128); 1 (32); 2 (8)	None	S80I	None	G103S	None
1 (128); 1 (64)	S83L, D87N	S80I	I355T,L416F	None	None
1 (128)	None	S80I	None	G103S	V29G
1 (64)	S83L, D87Y	S80I	None	G103S	None
1 (64)	S83L, D87H	None	None	G103S	None
1 (64)	S83L, D87N	None	None	D67N,G103S	None
1 (32)	S83L, D87Y	S80I	None	None	None
2 (32)	S83L	None	None	G103S	None
1 (32)	S83L	None	None	None	None
1 (32)	S83L, D87G	None	None	None	None
1 (16)	None	S80I, E84K	None	None	None
1 (16)	None	E84K	None	None	None
1 (1)	None	S80I	None	None	None
1 (32)1 (4); 1 (1)	None	None	None	G103S	None
mutation rate	52.70%	28.38%	2.70%	35.14%	1.35%

Note: *DANO* Danofloxacin, *NO* number

### Prevalence of plasmid-mediated Fluoroquinolone-resistance genes

**Fig. 2:**
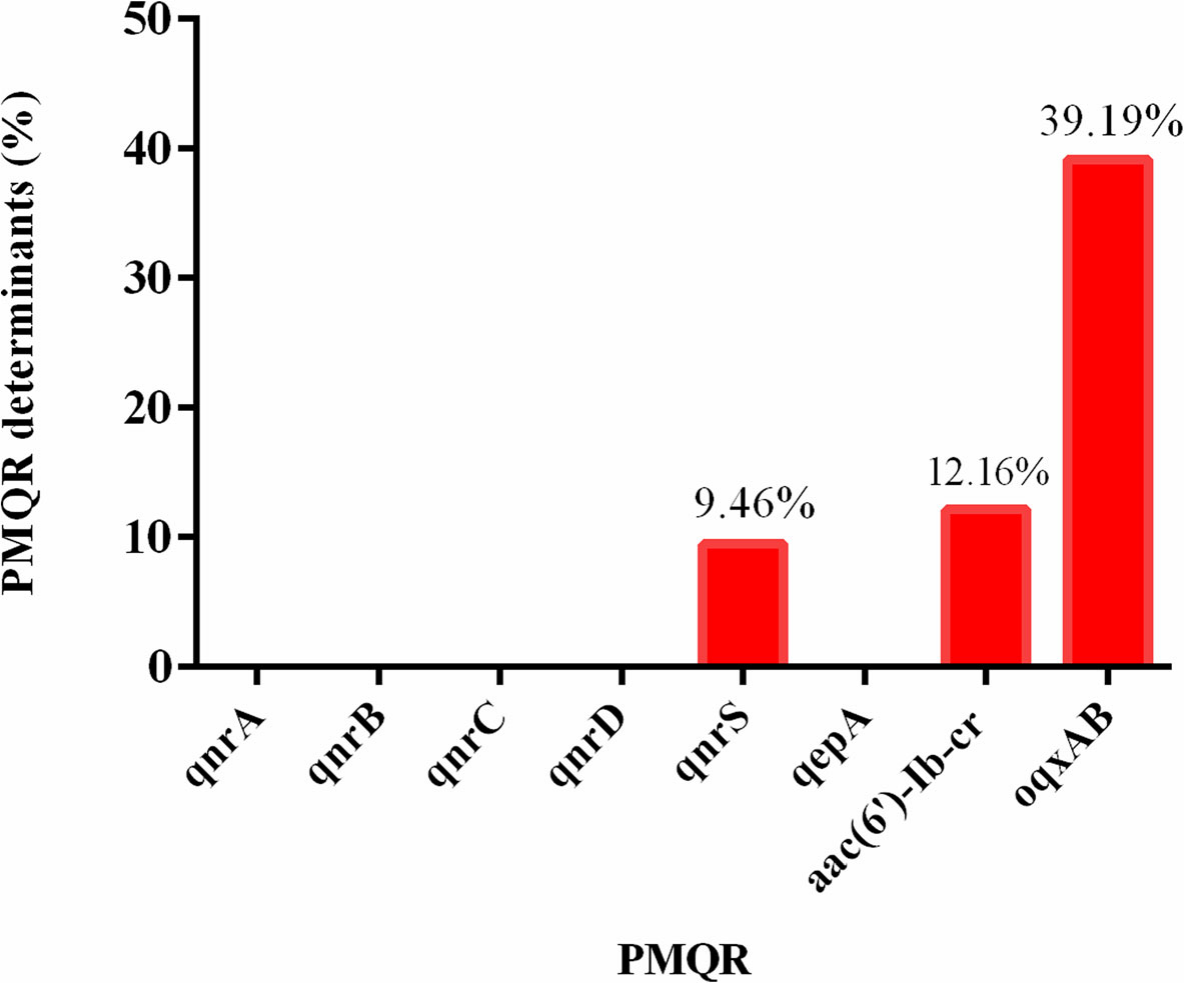
The prevalence of PMQR genes among 74 FQ-resistant *E. coli* isolates

As shown in Fig. 2, among the 74 *E. coli* isolates, *oqxAB* was the most prevalent PMQR genes, 29 (39.19%) strains carried the *oqxAB* gene. The number of isolates harbouring *qnrS* gene was 7 (9.46%). Four isolates (5.4%) were detected to co-harbour *oqxAB*, *qnrS* and *aac (6′)-Ib-cr* gene. One strains (1.4%) co-harbour *qnrS* and *oqxAB.* The number of strains co-harbour *oqxAB* and *aac (6′)-Ib-cr* was 2 (2.7%). Nine strains (12.16%) were positive for *aac (6′)-Ib-cr* gene. But no strains were found to be positive for *qnrA*, *qnrB*, *qnrC*, *qnrD* and *qepA.* The Gel electrophoresis of the PMQR genes was provided in Figure S2.

### Conjugative transfer of PMQR genes from swine farms

Conjugation experiments were carried out in this study to assess the transferability and the dissemination risk of PMQR genes in swine farm. The results of conjugation assays showed that *qnrS*, *oqxAB* and *aac (6′)-Ib-cr* could be successfully transferred to *E. coli* J53 from 4 (57.1%), 20 (69.0%) and 5 (55.6%) donor strains, respectively, at frequencies of 3.5 × 10^− 8^ to 4.7 × 10^− 5^, 2.1 × 10^− 9^ to 5.3 × 10^− 6^ and 1.8 × 10^− 8^ to 5.8 × 10^− 4^ transconjugants/recipient, respectively. The transconjugants were also FQ-resistant, but they showed low-level resistance to FQs antimicrobials as compared with donor strains.

## Discussion

The frequencies of resistance to FQs among *E. coli* strains isolated from humans and animals have been reported to be rising year by year in China, which might partly be attributable to the unreasonable and increasing use of this class of antimicrobial in food-producing animals [16, 17]. In the present study, the results of susceptibility test revealed that the 479 *E. coli* isolates collected from several swine farms in China and covering the MIC distribution range (0.0075- > 128 μg/mL) of DANO showed moderate rate of resistance to other four FQs antimicrobials (ciprofloxacin, ofloxacin, levofloxacin and norfloxacin). Further analysis showed that the percentage of strains resistant to the four FQs antimicrobials was positively correlated with the degree of resistance to DANO. In fact, ofloxacin and norfloxacin are not approved for veterinary uses, which are used in humans to prevent bacterial infection. But ciprofloxacin, norfloxacin and DANO have been used widely in veterinary medicine in China [18, 19]. It is well known that the indiscriminate use of FQs antimicrobials in humans and animals could raise a potential risk for the selection and acceleration the emergence of FQ resistance [4]. A previous report revealed very high rate of ciprofloxacin (75.2%), enrofloxacin (81.0%) and levofloxacin (70.5%) resistance in the *E. coli* isolated from diseased food-producing animals in Guangdong province, China [20]. The higher resistant rate could be due to the different origin of *E. coli* strains compared with this study.

The rapid development of FQ resistance is commonly acknowledged to be due to the widespread dissemination of PMQR determinants. Though PMQR determinants usually result in low-level resistance to FQs antimicrobials, the presence of PMQR determinants can promote and accelerate the occurrence of target mutations on the chromosome which mediate high-level resistance [21]. Some surveys have reported a high prevalence of PMQR determinants and occurrence of mutations in the QRDR among *E. coli* and *Salmonella* strains isolated from humans and food-producing animals in China [22, 23]. In this study, a high prevalence (60.8%) of PMQR determinants (*qnrS*, *oqxAB* and *aac (6′)-Ib-cr*) was found in the 74 FQ-resistant *E. coli*, and the most prevalent PMQR gene was *oqxAB* (39.19%), which were in line with the finding of a previous study [24]. In the present study, the *aac (6′)-Ib-cr* gene (12.16%) was also a prevalent PMQR gene, similarly to the result of a study from China in which *oqxAB* was not analyzed [25]. The *qnrS* (9.46%) was predominantly present among the *qnr*-type genes in this study, which was consistent with previous study [22, 25]. Our results supported previous findings that the prevalence of *aac (6′)-Ib-cr* was higher than that of *qnr* [26]. There were no *qnrA*, *qnrB*, *qnrC*, *qnrD* and *qepA* genes detected in this study.

The mutation frequency (95.9%) of the 74 FQ-resistant *E .coli* in this study was higher than that found in 14 *qnr*-positive *E. coli* strains isolated from farm animals (35.7%) [22, 27]. Similar to our findings, it was reported that 39 nalidixic acid-resistant *E. coli* strains isolated from diseased farm animals had a high mutation frequency (100%) [16, 28]. The different criteria used for isolates selection may be responsible for the differences among these studies. In this study, a total of 39 (52.7%) strains carried mutations in *gyrA*, including S83L and D87 to N or Y. These mutations at positions 83 and 87 have previously been detected in FQ-resistant *E. coli* strains [20]. Among the isolates with mutations, the most frequent mutations were at codons 83 and 87 in *gyrA* in the QRDR, and the most common type of amino acid substitution were S83L and D87N in *gyrA*, the results were consistent with previous study [29, 30]. It has been demonstrated that double-mutant (S83L and D87N) enzyme-DNA complexes had a lower affinity for FQs antimicrobials than wild-type complexes [13].

Though there were no mutations in *gyrB* among 74 FQ-resistant *E. coli* strains, we could not ignore the possibility of *gyrB* mutations in *E. coli* strains resistant to FQs, but previous study have also reported their absence [31]. Two different mutations were found in *parC*, including 24 (32.4%) strains carried mutations altering amino acid S80I and 3 (4.1%) strains carried E84K. The two mutations have been previously demonstrated to be associated with FQ resistance in *E. coli* [20]. Four strains had mutations in *parE* (I355T and L416F) which have been detected in FQ-resistant *E. coli* isolated from swine fecal samples in Korea [32]. Only two *parE* mutations, L445H and L416F have been proven to be associated with FQ resistance in *E. coli* [14, 33]. To the best of our knowledge, L416F mutation has never been reported in China.

It has been demonstrated that the mutations in the repressor proteins marR and acrR can result in FQ-resistance by promoting the expression of the AcrAB-TolC efflux pump [11]. In the present study, a total of 26 strains with mutations in *marR*, 25 strains of those carried single mutation (G103S) and 1 strain of those carried two mutations (D67N and G103S). It has been reported that no significant association was found between the mutation *marR*-G103S and any level of FQ resistance [14]. The mutations in the region of *marR* (D76G, L78M, and V79I) have been reported to result in FQ resistance [11]. The possible role of novel mutation *marR*-D67N in FQ-resistant isolate requires further study to reveal. Only one strain carried mutation *acrR*-V29G which was also detected among highly levofloxacin-resistant *E. coli* isolates in previous study. The possible roles of *acrR*-V29G in FQ resistance may contribute to increased fitness rather than increased levels of resistance [14]. Moreover, some studies have reported that the coexistence of *acrR* gene mutations and other known chromosomal mutations in clinical strains of *E. coli* can lead to high-level FQ resistance [34].

## Conclusion

In summary, DANO-resistant *E. coli* strains isolated from swine farms had significant cross-resistance to other four FQs antimicrobials, and the percentage of strains resistant to other FQs antimicrobials was positively correlated with the degree of resistance to DANO. Furthermore, the resistance mechanisms of swine-derived *E. coli* to FQs may be attributable to the occurrence of chromosomal mutations (*gyrA*, *parC*, *parE*, *marR* and *acrR* genes double-site or single-site mutation) and the presence of PMQR determinants (*qnrS, oqxAB and aac (6′)-Ib-cr*). To the best of our knowledge, one novel mutation *marR*-D67N was found to be associated with FQ resistance, and two mutations *parE*-L416F and *acrR*-V29G have never been reported in China.

## Methods

### Sampling and bacterial isolates

From July, 2014 to March, 2017, a total of 300 piggery wastewater samples were collected from 21 swine farms located in different geographic areas of China, including Heilongjiang, Jilin, Liaoning, Henan, Hubei, Shandong and Yunnan province (Table 1). The samples brought to the laboratory were immediately cultured on MacConkey agar at 37 °C for 18–24 h, and then five colonies with typical *E. coli* morphology were selected from each sample. The bacterial strains were identified using classing biochemical methods and confirmed as *E. coli* by PCR amplification of *16S rRNA* and sequencing. In addition, a total of 1000 *E. coli* strains were selected from our previous study. All confirmed *E. coli* isolates were stored at − 80 °C for further studies.

### Antimicrobial susceptibility testing

For all isolated *E. coli*, the microdilution broth method in accordance with the guidelines in Clinical and Laboratory Standards Institute (CLSI) document M07-A9 was performed to determine the MICs of DANO. Moreover, the results were interpreted according the breakpoint which was established in the previous study [5]. The antimicrobial susceptibilities of *E. coli* to ciprofloxacin, ofloxacin, levofloxacin and norfloxacin were determined by disk diffusion method described by European Committee on Antimicrobial Susceptibility Testing (EUCAST). The interpretation of the result was according to EUCAST criteria.

### Screening for Fluoroquinolone resistance-associated mutations

DNA templates of the FQ-resistant *E. coli* isolates were obtained with the Kit (Tiangen) following the manufacturer’s instructions. Screening for mutations accounting for FQ resistance including *gyrA*, *gyrB*, *parC*, *paE, marR* and *acrR* was carried out by PCR amplification and DNA sequencing*.* The oligonucleotide primers used, together with details of the specific regions sequenced in each gene were the same as previously described [11]. The amplification products were visualized by agarose gel electrophoresis and ethidium bromide staining to assess the sizes of the gene fragments, and then the positive products were validated with Sanger sequencing. The obtained sequences were analyzed with the Chromas and the amino acid sequences of *gyrA*, *gyrB*, *parC*, *paE, marR* and *acrR* were compared with wild-type *E. coli* K-12 to determine the amino acid changes. Besides, the Sorting Intolerant From Tolerant (SIFT) scores (http://sift.jcvi.org) were calculated by online software to determine whether amino acid changes in *gyrA*, *gyrB*, *parC*, *paE, marR* and *acrR* affect protein function. (neutral or deleterious).

### Screening for plasmid-mediated Fluoroquinolone-resistance determinants

Plasmid DNA of the resistant *E. coli* isolates was obtained with plasmid extraction kit following the manufacturer’s instructions. Multiplex PCR was performed for the detection of PMQR genes, including *qnrA, qnrB, qnrS, qepA, oqxAB, qnrC, qnrD* and *aac (6′)-Ib-cr*. The oligonucleotide primers and the details of PCR amplification were used as previously described [35].

### Conjugative transfer of PMQR genes

For assessing the transferability of the PMQR genes in swine farms, conjugation experiment was performed according to method described by Ghosh and Mukherjee [36]. The strains of *qnrS*-positive *E. coli*, *oqxAB*-positive *E. coli*, and a *aac (6′)-Ib-cr*-positive *E. coli* isolates were used as the donors of PMQR genes in conjugation experiments, and *E. coli* J53 strains with azide resistance was used as recipient. The MacConkey agar plates containing sodium azide (100 μg/L) and ciprofloxacin (5 μg/mL) were used to select PMQR genes positive transconjugants. PCR analysis and DNA sequencing were carried out to confirm that transconjugants were derivatives of the recipient strain *E. coli* J53. The transfer frequencies of *qnrS*, *oqxAB* and *aac (6′)-Ib-cr* genes were determined as described in a previous study [37].

### Statistical analysis

Descriptive analyses on percentage and prevalence were performed using functions provided in Excel 2007 (Microsoft Software). To determine the correlation of the resistance rate of *E. coli* to four FQs antimicrobials with the DANO MIC values, Pearson correlation and linear regression analysis were performed by using GraphPad Prism 6 for windows. *P-*value less than 0.05 was considered statistically significant.

## Supplementary information

**Additional file 1: Table S1.** The area distribution of collected swine *E. coli* isolates. **Table S2.** Putative FQ resistance mutations and the prevalence of PMQR genes in swine-derived *E. coli.***Figure S1.** Gel electrophoresis of the FQ-resistance associated gene on the chromosome in swine-derived *E. coli.***Figure S2.** Gel electrophoresis of the PMQR genes in swine-derived *E. coli.*

## Data Availability

The datasets used and analyzed in this study are available from the corresponding author on reasonable request.

## Abbreviations

FQs: Fluoroquinolones
ARB: Antimicrobial-resistant bacteria
PMQR: Plasmid-mediated quinolone resistance
*E. coli*: *Escherichia coli*
DANO: Danofloxacin
QRDR: Fluoroquinolone resistance determining region
CLSI: Clinical and Laboratory Standards Institute
EUCAST: European Committee on Antimicrobial Susceptibility Testing
SIFT: Sorting Intolerant From Tolerant
