# Supplementary material for: The prevalence and mechanism of fluoroquinolone resistance in Escherichia coli isolated from swine farms in China
Source: BMC Vet Res. 2020 Jul 28;16:258. doi: 10.1186/s12917-020-02483-4 (PMC7388466; doi:10.1186/s12917-020-02483-4)
Supplement: Supplementary file 1 — Additional file 1: Table S1. The area distribution of collected swine E. coli isolates. Table S2. Putative FQ resistance mutations and the prevalence of PMQR genes in swine-derived E. coli.Figure S1. Gel electrophoresis of the FQ-resistance associated gene on the chromosome in swine-derived E. coli.Figure S2. Gel electrophoresis of the PMQR genes in swine-derived E. coli. [file 12917_2020_2483_MOESM1_ESM.doc]

Table S1 The area distribution of collected swine *E. coli* isolates

| Farm location | No of isolated *E. coli* strains | No of strains for AST to FQ |
| --- | --- | --- |
| Heilongjiang | 404 | 126 |
| Jilin | 263 | 104 |
| Liaoning | 227 | 96 |
| Shandong | 30 | 21 |
| Henan | 249 | 96 |
| Hubei | 20 | 20 |
| Yunnan | 29 | 16 |

Note：NO, number; AST, Antimicrobial Susceptibility Testing; FQ, fluoroquinolone

Table S2 Putative FQ resistance mutations and the prevalence of PMQR genes in swine-derived *E. coli*

| Strain | Target mutation | | | | | | PMQR | | | | | | |
| --- | --- | --- | --- | --- | --- | --- | --- | --- | --- | --- | --- | --- | --- |
|  | DAN MIC | *gyrA* | *parC* | *parE* | *marR* | | | *acrR* | *qnrS* | | | *oqxAB* | *aac (6')-Ib-cr* |
| HP59 | >128 | S83L, D87N | - | - | - | | | - | - | | | - | - |
| HP61 | >128 | S83L, D87N | S80I | - | G103S | | | - | - | | | **+** | **+** |
| HP69 | >128 | S83L, D87N | - | - | - | | | - | - | | | - | - |
| HP79 | >128 | S83L, D87N | - | - | - | | | - | - | | | - | - |
| HP171 | >128 | S83L, D87N | - | - | - | | | - | - | | | - | - |
| HP313 | >128 | S83L, D87N | - | - | - | | | - | - | | | - | - |
| HP314 | >128 | S83L, D87N | - | - | - | | | - | - | | | **+** | - |
| HP327 | >128 | S83L, D87N | S80I | - | - | | | - | - | | | - | - |
| HP460 | >128 | S83L, D87N | S80I | - | G103S | | | - | - | | | - | - |
| HP464 | >128 | S83L, D87N | - | - | G103S | | | - | - | | | - | **+** |
| HP465 | >128 | S83L, D87N | S80I | - | - | | | - | - | | | - | - |
| HP466 | >128 | S83L, D87N | - | - | - | | | - | **+** | | | **+** | - |
| HP473 | >128 | - | S80I | - | G103S | | | - | - | | | - | - |
| HP485 | >128 | S83L, D87N | - | - | - | | | - | - | | | - | - |
| HP491 | >128 | S83L, D87N | - | - | - | | | - | - | | | - | - |
| HP503 | >128 | S83L, D87N | - | - | - | | | - | - | | | - | - |
| HP8 | 128 | S83L, D87N | - | - | G103S | | | - | - | | | **+** | - |
| HP63 | 128 | S83L, D87N | S80I | - | G103S | | | - | - | | | **+** | **+** |
| HP204 | 128 | S83L, D87N | S80I | I355T, L416F | - | | | - | **+** | | | **+** | **+** |
| HP217 | 128 | S83L, D87N | - | - | - | | | - | - | | | **+** | - |
| HP337 | 128 | - | S80I | - | G103S | | | V29G | | | - | - | **+** |
| HP441 | 128 | S83L, D87N | - | - | - | | | - | | **+** | | - | - |
| HP478 | 128 | S83L, D87N | S80I | - | G103S | | | - | | - | | - | - |
| HP494 | 128 | S83L, D87N | S80I | - | G103S | | | - | | - | | **+** | - |
| HP502 | 128 | S83L, D87N | - | - | - | | | - | | - | | - | - |
| HP504 | 128 | - | S80I | - | G103S | - | | | | - | | - | - |
| HP226 | 64 | - | - | - | - | | | - | | **+** | | **+** | **+** |
| HP229 | 64 | S83L, D87N | S80I | I355T, L416F | - | | | - | | **+** | | **+** | **+** |
| HP336 | 64 | S83L, D87N | - | - | D67N,  G103S | | | - | | **+** | | **+** | **+** |
| HP359 | 64 | S83L, D87N | S80I | - | G103S | | | - | | - | | **+** | - |
| HP436 | 64 | S83L, D87Y | S80I | - | G103S | | | - | | - | | **+** | - |
| HP438 | 64 | S83L, D87N | S80I | - | G103S | | | - | | - | | + | - |
| HP456 | 64 | S83L, D87N | S80I | - | G103S | | | - | | - | | + | - |
| HP457 | 64 | S83L, D87H | - | - | G103S | | | - | | - | | + | - |
| HP470 | 64 | S83L, D87N | - | - | - | | | - | | - | | - | - |
| HP475 | 64 | S83L, D87N | - | - | - | | | - | | - | | - | - |
| HP481 | 64 | S83L, D87N | S80I | - | - | | | - | | - | | - | - |
| HP2 | 32 | S83L, D87Y | S80I | - | - | | | - | | - | | + | - |
| HP67 | 32 | - | S80I | - | G103S | | | - | | - | | - | - |
| HP75 | 32 | S83L | - | - | G103S | | | - | | - | | - | - |
| HP76 | 32 | S83L | - | - | - | | | - | | - | | - | - |
| HP84 | 32 | S83L, D87G | - | - | - | | | - | | - | | - | - |
| HP181 | 32 | S83L | - | - | G103S | | | - | | - | | + | - |
| HP182 | 32 | - | - | - | G103S | | | - | | - | | + | - |
| HP189 | 32 | S83L, D87N | - | - | G103S | | | - | | - | | + | - |
| HP39 | 16 | - | S80I, E84K | - | - | | | - | | - | | + | - |
| SDP4 | 16 | - | E84K | - | - | | | - | | - | | + | - |
| SDP6 | 16 | - | - | - | - | | | - | | - | | + | - |
| SDP11 | 16 | - | - | - | - | | | - | | - | | + | - |
| SDP13 | 16 | - | S80I, E84K | - | G103S | | | - | | - | | + | - |
| LNP21 | 8 | - | S80I | - | G103S | | | - | | - | | - | - |
| LNP26 | 8 | - | S80I | - | G103S | | | - | | - | | - | - |
| LNP60 | 8 | - | - | - | - | | | - | | + | | - | - |
| HP206 | 4 | - | - | - | G103S | | | - | | - | | + | - |
| HP198 | 1 | - | - | - | G103S | | | - | | - | | + | - |
| HP201 | 1 | - | S80I | - | - | | | - | | - | | + | + |
| SDP14 | 0.5 | - | - | - | - | | | - | | - | | + | - |


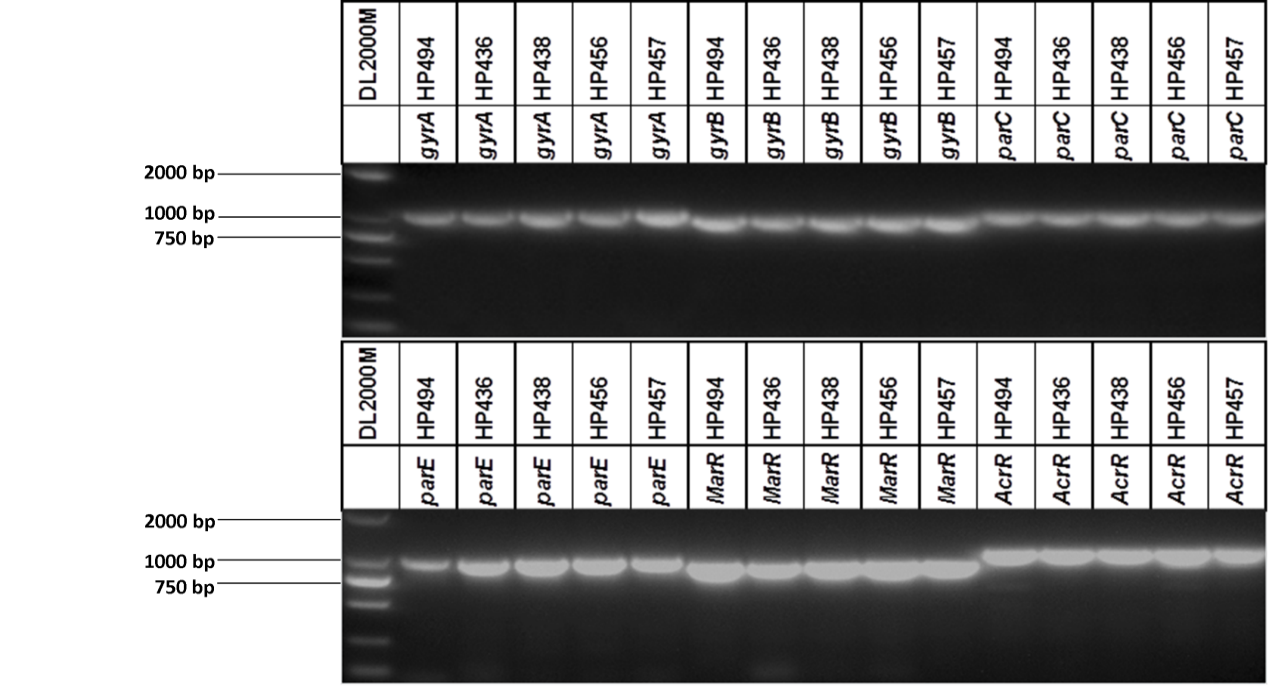


Figure S1 Gel electrophoresis of the FQ-resistance associated gene on the chromosome in swine-derived *E. coli*


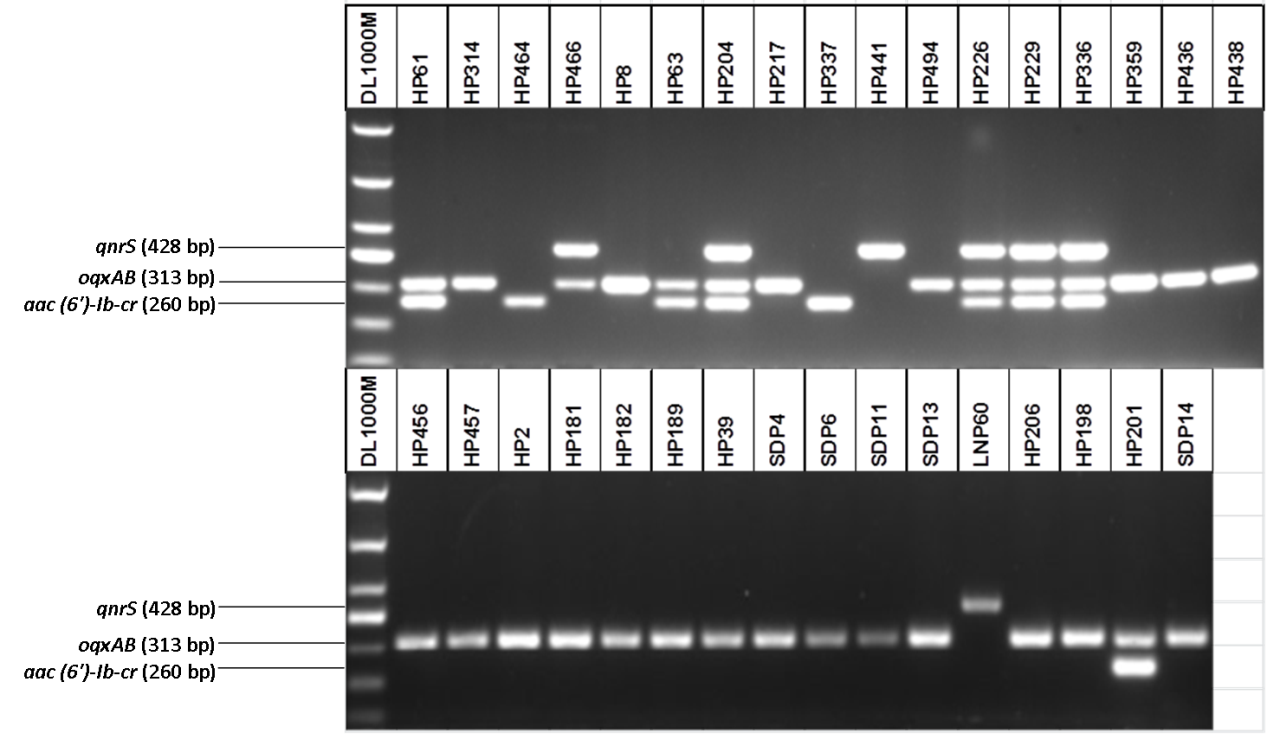


Figure S2 Gel electrophoresis of the PMQR genes in swine-derived *E. coli*
